# Genomic and phenotypic diversity among taxonomically ambiguous clinical *Corynebacterium* isolates

**DOI:** 10.1186/s12866-025-04619-8

**Published:** 2025-12-22

**Authors:** Holger Brüggemann, Lise Hald Schultz, Anja Poehlein, Bo Söderquist

**Affiliations:** 1 Department of Biomedicine, Faculty of Health, Aarhus University, Aarhus, Denmark; 2 School of Medical Sciences, Faculty of Medicine and Health, Örebro University, Örebro, Sweden; 3 Department of Genomic and Applied Microbiology, Institute of Microbiology and Genetics, University of Göttingen, Göttingen, Germany; 4 Department of Orthopedics, Faculty of Medicine and Health, Örebro University, Örebro, Sweden

**Keywords:** Corynebacterium, *Corynebacterium tuberculostearicum* complex, *Corynebacterium kroppenstedtii* complex, Corynebacterial infections, Antimicrobial resistance

## Abstract

**Background:**

*Corynebacterium* is a widespread and abundant bacterial genus on human skin. Occasionally, corynebacteria are isolated from clinical specimens associated with infection. In this study, 56 bacterial isolates were examined. These isolates were obtained from 52 patients with diverse infections such as keratitis, osteitis/osteomyelitis, mastitis, (suspected) foreign body associated infections (spine, prosthetic joint), suspected meningitis, post-operative infections, among others. These isolates were identified as corynebacteria by MALDI-TOF mass spectrometry but could not be reliably assigned to a specific species. To resolve this issue, the isolates were genome-sequenced, and species identification was done with different approaches, including digital DNA–DNA hybridization, phylogenomic tree placement and Average Nucleotide Identity (ANI) calculations. A subset of 34 strains was further investigated by biochemical characterization and antimicrobial susceptibility testing (AST).

**Results:**

The 56 isolates belonged to 28 distinct corynebacterial species. Species identification was particularly ambiguous for 13 isolates as the ANIs were below 95% to the closest identified reference genomes. Two isolates represented potentially novel species, since no close relative could be identified (ANI < 90%). The majority of isolates belonged to the *Corynebacterium marquesiae/tuberculostearicum* (*n* = 10) and *Corynebacterium kroppenstedtii/parakroppenstedtii* (*n* = 9) complexes. Biochemical tests and AST revealed species- and strain-level variability. AST demonstrated extensive antimicrobial resistance (AMR), particularly among *C. marquesiae*,* C. tuberculostearicum*, *C. lehmanniae*, *C. hesseae* and *C. resistens*, with resistances observed against penicillin, clindamycin, ciprofloxacin, and rifampin. Resistance was frequently associated with acquired AMR genes, such as *erm(X)*,* tet(W)* and genes encoding aminoglycoside-modifying enzymes. Among the tested antibiotics, clindamycin resistance was most common, detected in 23 of 34 tested strains (64.7%).

**Conclusions:**

This study expands our knowledge of *Corynebacterium* isolates derived from clinical specimens, particularly those differing from well-characterized species. It underscores the extensive geno- and phenotypic variability within most *Corynebacterium* species and challenges current species boundary definitions. The extensive level of detected AMR may complicate treatment of underlying infections. However, it remains uncertain whether these isolates represent true infectious agents or contaminants derived from the skin of the patients.

**Supplementary Information:**

The online version contains supplementary material available at 10.1186/s12866-025-04619-8.

## Background

*Corynebacterium* spp. are widely distributed in nature and in humans, particularly as part of the normal human skin microbiota [1–3]. The genus comprises more than 130 species, with recently identified novel species among cutaneous and nasal corynebacteria [4–7]. A few *Corynebacterium* species are considered pathogens, including *Corynebacterium diphtheriae*, the primary cause of diphtheria, and related species, such as *Corynebacterium ulcerans* and *Corynebacterium pseudotuberculosis* [8, 9].

Infections in humans caused by non-diphtherial corynebacteria are relatively rare and have been mainly reported for opportunistic and/or nosocomial infections [10, 11]. Risk groups include immunocompromised patients, preterm infants, and patients with indwelling medical devices. Reported infections include bacteraemia/sepsis, urinary tract infections (UTIs), catheter-related bloodstream infections, respiratory infections, osteomyelitis, infective endocarditis, keratitis, wound and surgical site infections (SSIs) and device-related infections, such as prosthetic joint infections (PJIs) [12–15]. Among prominent non-diphtherial corynebacterial species that cause infections are *Corynebacterium urealyticum*, causing mainly UTIs, *Corynebacterium striatum*, causing diverse nosocomial infections, and *Corynebacterium macginleyi*, causing keratitis [16–21]. There are also case reports regarding isolates belonging to other corynebacterial species, such as *Corynebacterium jeikeium*,* Corynebacterium amycolatum*,* Corynebacterium aurimucosum*,* Corynebacterium kroppenstedtii*, *Corynebacterium pseudodiphtheriticum*,* Corynebacterium propinquum*, *Corynebacterium accolens* and others [10, 11, 22–24]. Multidrug resistance has been found in several clinical isolates, particularly in *C. jeikeium* and *C. striatum*, complicating treatment [25–28].

The diagnosis of corynebacteria as an etiological agent of the disease remains challenging for several reasons. As corynebacteria are prominent and omnipresent members of the normal skin microbiota, their isolation from clinical specimens can represent contamination. Specific criteria are often used to distinguish between infection causality and contamination. For instance, in the case of PJI, such criteria include repeated isolation from PJI tissue specimens (i.e. growth from multiple periprosthetic tissue samples), clinical signs of infection, inflammatory markers, and/or histopathological evidence [29]. Other diagnostic challenges are related to the fastidious growth and growth differences between corynebacterial species [1]. Some species are slow-growing and may be outcompeted in mixed cultures, thus leading to false-negative cultures. Regarding accurate species identification, traditional phenotypic methods, such as biochemical tests often misidentify or lump *Corynebacterium* spp. together, as they can be phenotypically very similar. Tools such as MALDI-TOF mass spectrometry (MS) and 16S rRNA sequencing have drastically improved the accuracy and speed of corynebacterial species identification in clinical laboratories [13, 30, 31]. However, challenges remain due to the ongoing discovery of novel corynebacteria [4–7], which means that corynebacterial sequence/proteome databases are still incomplete.

This study aimed to gather knowledge on the diversity and properties of corynebacterial clinical isolates that could not be unambiguously assigned to a specific species by MALDI-TOF MS. Genome sequencing, biochemical assays, and drug susceptibility testing were performed to obtain a broad overview of their genotypes and phenotypes.

## Methods

### Strain cohort

The strain collection database at the Department of Laboratory Medicine, Clinical Microbiology, Örebro University Hospital, Örebro, Sweden, was searched for corynebacterial strains that were isolated from clinical specimens. Most clinical specimens were taken from usually sterile body sites, e.g., blood culture, deep tissue biopsies (periprosthetic tissue, mediastinal tissue, lung tissue), cerebrospinal fluid, synovial fluid and peritoneal fluid. Other specimens were taken from sites where commensals can be (occasionally) cultivated as well, such as corneal swab, wound swab/surgical site/pus, middle ear exudate, breast abscess fluid and breast milk. Corynebacteria/coryneform bacteria were the predominant organisms found upon cultivation of the included clinical specimens. Only strains were further included in this study that were assigned as *Corynebacterium* spp. by MALDI-TOF MS (Microflex LT and Biotyper 3.1, Bruker Daltonik, Bremen, Germany) but could not be further assigned to a specific corynebacterial species. This non-assignment was due to low identification scores obtained by MALDI-TOF MS, with scores < 1.7. In total, 56 corynebacterial isolates were randomly selected from the strain collection.

### Cultivation

Isolates were stored in preservation medium (trypticase soy broth with 0.3% yeast extract and 29% horse serum) at − 80 °C and were for the present study initially inoculated on GC agar (GC Medium Base, Becton Dickinson (BD), Sparks, Maryland, USA, supplemented with 1% BBL IsoVitaleX enrichment), and incubated in an aerobic atmosphere containing 5% CO_2_ at 36 °C. For further cultivation, FTO (Furazolidone, Tween-80, Oil red O) agar medium was used [32] with the following composition: 40 g trypticase soy agar (TSA), 5 g yeast extract, 10 mL Tween-80, 1 L ultra-filtrated-water; after autoclaving furazolidone (6 µg/mL) and 1 mL of Oil red O (0.5% stock solution) were added. FTO agar plates were incubated in an aerobic atmosphere containing 5% CO_2_ at 36 °C for up to six days.

### Genomic DNA extraction

The MasterPure™ Gram Positive DNA Purification Kit (Lucigen) was used according to the manufacturer’s instructions. DNA quality and yield was checked by agarose gel electrophoresis along with concentration determination using the Qubit^®^ dsDNA HS Assay Kit (Life Technologies GmbH, Darmstadt, Germany).

### Genome sequencing

Illumina shotgun libraries were prepared using the Nextera XT DNA Sample Preparation Kit and subsequently sequenced (paired-end; 2 × 300 bp) on a MiSeq system using the v3 reagent kit with 600 cycles (Illumina, San Diego, CA, United States) as recommended by the manufacturer. Quality filtering was done with version 0.39 of Trimmomatic [33]. Assembly was performed with version 3.15.2 of the SPAdes genome assembler software [34]. Version 2.2.1 of Qualimap was used to validate the assembly and determine the sequence coverage [35]. Default parameters were used for all mentioned software unless otherwise specified. In total, 56 corynebacterial strains were sequenced with a genome coverage of 76- to 411-fold (in average 223-fold) (Table S1). All 56 draft genome sequences were deposited in GenBank (accession number PRJNA1327770).

### Bioinformatics tools

Gene prediction and annotation of all genomes were performed with RAST [36]. Blastn was used to find the closest relative of all 56 sequenced genomes, using the NCBI databases core nucleotide database (core_nt), RefSeq reference genomes, RefSeq genome database and whole-genome shotgun contigs (status April 2025). For further species identification, these tools were used: Type (Strain) Genome Browser (TYGS; https://tygs.dsmz.de/) which implements established taxonomy methods such as Genome-to-Genome Distance Calculator (GGDC) and Genome BLAST Distance Phylogeny (GBDP)) [37]; Ribosomal Multilocus Sequence Typing (rMLST; https://pubmlst.org/species-id) [38] and Genome Taxonomy Database toolkit (GTDB-tk, v.2.5.2) [39]. JSpeciesWS was used for average nucleotide identity (ANI; ANIb, based on BLAST) calculations [40]. Reference genomes from the closest corynebacterial relatives identified by the above-mentioned methods were used along with the 56 corynebacterial genomes from this study to build a core genome-based phylogeny using CSI phylogeny [41]. Here, the phylogenetic tree was constructed using an approximate Maximum Likelihood method implemented in FastTree [42]. Phylogenetic trees were visualized using the Interactive Tree of Life [43]. For the detection of reliable core genome single-nucleotide variants, the Parsnp program from the Harvest software package was used [44]. ResFinder (http://genepi.food.dtu.dk/resfinder) was used to analyze genomes for antimicrobial resistance determinants [45].

### Biochemical tests

Enzymatic activities and fermentation abilities of 34 selected corynebacterial strains were tested with the API^®^ CORYNE system (Biomerieux). The system comprised 19 tests, including 11 tests for enzymatic activities. These activities included nitrate reduction, pyrazinamidase, pyrrolidonyl-arylamidase, alkaline phosphatase, β-glucosidase, β-glucuronidase, β-galactosidase, α-glucosidase, N-acetyl-β-glucosaminidase, urease and hydrolysis of gelatin. Fermentation of eight sugars, namely glucose, ribose, xylose, mannitol, maltose, lactose, sucrose and glycogen were tested. The tests were carried out following the instructions of the manufacturer. Bacterial suspensions with a turbidity greater than 6 McFarland were prepared, which were then used to inoculate the 11 enzymatic tests. For the fermentation tests, 0.5 mL of the suspension was transferred to the API^®^ GP medium, and the suspension was added to the last nine wells on the strip. The fermentation wells and the urea well were sealed with mineral oil. The strips were incubated under aerobic conditions for 24 h at 37 °C. Subsequently, specific reagents were added to the wells according to the manufacturer instructions and results were recorded after 10 min. The API^®^ CORYNE test was done in triplicates. When results differed between replicates or when the color could not be clearly interpreted as positive or negative, results were noted as inconclusive.

### Antimicrobial susceptibility testing

Antimicrobial susceptibility testing (AST) was done to evaluate the susceptibility of 34 selected corynebacterial strains to a panel of commonly used antibiotics, namely penicillin, ciprofloxacin, clindamycin, vancomycin and rifampicin. The disc diffusion assay (DDA) was applied according to EUCAST instructions (https://www.eucast.org/). In brief, bacterial suspensions with a turbidity of 0.5 McFarland were prepared. The EUCAST guidelines use Mueller-Hinton agar + 5% defibrinated horse blood and 20 mg/L β-NAD (MH-F). In contrast to FTO plates, poor growth on MH-F plates of several species/isolates was noticed. Zone diameters on FTO and MH-F plates were compared for eight isolates; they were largely comparable for those isolates that grew on both plates. DDA was thus performed on FTO agar plates. After inoculation of the plates and a 15 min drying time, the antibiotic discs were gently pressed onto the plates using a sterile tweezer. The plates were then incubated for 20–44 h in an aerobic atmosphere containing 5% CO_2_. The DDA was done in triplicates. The EUCAST table “*Corynebacterium* spp. other than *C. diphtheriae* and *C. ulcerans*” (version 15.0, valid from 2025-01-01) was used to read the zone diameter breakpoints.

## Results

### Selection of corynebacterial isolates from clinical specimens

In the present study, 56 corynebacterial clinical isolates that could not be assigned to the species level due to a low MALDI TOF MS identification score (score < 1.7) were randomly selected from the strain collection at the Department of Laboratory Medicine, Clinical Microbiology, Örebro University Hospital, Örebro, Sweden. These 56 isolates were derived from 52 patients. The bacteria were isolated from diverse clinical specimens associated with various infections including keratitis (11 patients); SSI (8 patients); osteitis/osteomyelitis (7 patients); postoperative spinal implant infection (7 patients); PJI (6 patients); mastitis (4 patients), and other infections/specimens (cerebrospinal fluid, lung biopsy, mastoiditis, ascites, prosthetic valve endocarditis) (Table 1).

**Table 1:** Taxonomy assignment and genome sequence statistics of 56 corynebacterial isolates and their clinical origin

strain name	infection/clinical specimen/site	assigned species	TYGS	rMLST (% identity)	GTDB-Tk	ANI reference genome (%)	GC (%)	Size (bp)
22–2113	PJI (shoulder)	*pilbarense*	*pilbarense*	*pilbarense* (73)	*pilbarense*	97.16	65	2,371,572
21–884	Osteitis (os ilium)	aurimucosum *	*aurimucosum*	*aurimucosum* (78)	*aurimucosum*	92.54	60.5	2,681,163
23–416	Keratitis	*bovis*	bovis	*bovis* (100)	*bovis*	98.26	72.8	2,577,215
23–137	Keratitis	*bovis*	bovis	*bovis* (100)	*bovis*	98.27	72.8	2,562,342
23–1351	SSI, achilles tendon surgery	confusum *	PNS	*confusum* (28)	sp001815935	94.46	65.2	2,455,975
18–4917	Osteitis (hand fracture)	confusum *	PNS	*confusum* (28)	sp001815935	94.07	65.1	2,402,416
22–2107	Prosthetic valve endocarditis	*coyleae*	*coyleae*	*coyleae* (100)	*coyleae*	97.33	61.5	2,478,179
21–258	Ascites (liver cirrhosis)	*hesseae*	*hesseae*	*hesseae* (78)	*hesseae*	96.79	61.2	2,676,028
23–692	SSI, post inguinal hernia surgery	*hesseae*	*hesseae*	*“guaraldiae”* (53)	*hesseae*	96.03	60.7	2,912,422
22–367	PJI (knee)	*gottingense*	*gottingense*	*gottingense* (38)	*gottingense*	96.74	65.2	2,680,382
19–2054	Mastitis	*parakroppenstedtii*	*parakroppenstedtii*	*parakroppenstedtii* (100)	*parakroppenstedtii*	99.94	56.8	2,562,819
22–714	Keratitis	kroppenstedtii *	PNS	*kroppenstedtii* (3)	*kroppenstedtii*_B	92.15	56.8	2,460,667
21–990	CSF, shunt infection	kroppenstedtii *	PNS	*kroppenstedtii* (11)	sp943913165	93.17	57.2	2,413,484
21–1619	Same patient as above	kroppenstedtii *	PNS	*kroppenstedtii* (11)	sp943913165	93.24	57.2	2,413,426
16–2461	Mastitis	*parakroppenstedtii*	*parakroppenstedtii*	*parakroppenstedtii* (100)	*parakroppenstedtii*	99.97	56.9	2,527,917
18–7157	Mastitis	*parakroppenstedtii*	*parakroppenstedtii*	*parakroppenstedtii* (100)	*parakroppenstedtii*	99.50	56.9	2,591,472
23–1844	PSII	kroppenstedtii *	PNS	*kroppenstedtii* (13)	sp943913165	93.18	57.2	2,413,757
18–1395	Mastitis	*parakroppenstedtii*	*parakroppenstedtii*	*parakroppenstedtii* (100)	*parakroppenstedtii*	99.51	56.9	2,581,496
23–3061	SSI, mediastinitis, cardiac surgery	parakroppenstedtii *	PNS	*parakroppenstedtii* (97)	sp902373425	91.80	56.1	2,442,675
22–331	CSF, post-traumatic meningitis	*lehmanniae*	PNS	*lehmanniae* (89)	*lehmanniae*	95.81	65.1	2,616,492
18–1686	Lung biopsy	*lehmanniae*	PNS	*lehmanniae* (56)	*lehmanniae*	95.60	65.4	2,439,088
17–4208	Keratitis	*macginleyi*	*macginleyi*	*macginleyi* (100)	*macginleyi*	98.02	57.1	2,389,627
22–800	Keratitis	*mastitidis*	*mastitidis*	*mastitidis* (100)	*mastitidis*	96.58	68.7	2,300,647
16–4937	Keratitis	*mastitidis*	*mastitidis*	*mastitidis* (100)	*mastitidis*	96.68	68.5	2,356,889
22–3933	Keratitis	*mastitidis*	*mastitidis*	*mastitidis* (100)	*mastitidis*	96.68	68.6	2,332,363
23–539	Keratitis	*mastitidis*	*mastitidis*	*mastitidis* (100)	*mastitidis*	96.62	68.6	2,349,709
20–2095	SSI, cardiac surgery	minutissimum *	PNS	*minutissimum* (100)	sp001812805	92.53	59.8	2,759,884
23–3035	SSI, mediastinitis, cardiac surgery	*“phoceense”*	*phoceense*	*phoceense* (100)	*phoceense*	99.33	63.4	2,729,481
21–2185	Osteomyelitis (tibia)	*“phoceense”*	*phoceense*	*phoceense* (100)	*phoceense*	99.31	63.4	2,729,546
16–7020	PSII	*pyruviciproducens*	*pyruviciproducens*	*pyruviciproducens* (100)	*pyruviciproducens*	97.26	60.8	2,756,098
15–3615	SSI, osteitis (foot fracture)	*resistens*	*resistens*	*resistens* (55)	*resistens*	97.07	57.1	2,568,614
17–2957	Mastoiditis	*riegelii*	*riegelii*	*riegelii* (96)	*riegelii*	96.83	60.5	2,530,533
22–2426	Keratitis	*sanguinis*	*sanguinis*	*sanguinis* (97)	*sanguinis*	96.96	65.2	2,395,476
22–1407	Lung biopsy	*sanguinis*	*sanguinis*	*sanguinis* (97)	*sanguinis*	97.70	65.3	2,297,927
23–1160	CSF, suspected neonatal meningitis	*sanguinis*	*sanguinis*	*sanguinis (100)*	*sanguinis*	97.29	65.4	2,359,489
22–193	Lung biopsy	*accolens*	*accolens*	*accolens* (95)	*accolens*	97.01	59.6	2,423,638
23–135	SSI, foot fraction	*accolens*	*accolens*	*accolens* (92)	*accolens*	96.94	59.6	2,402,137
17–2705	Keratitis	*rhinophilum*	*rhinophilum*	*accolens* (100)	sp943914355	97.86	59.0	2,503,480
20–2016	SSI, mastectomy	*simulans*	*simulans*	*simulans* (100)	*simulans*	98.71	59.3	2,518,822
22–2729	Osteomyelitis, foot fracture	novel species **	PNS	*jeikeium* (30)	sp946221935	-	59.3	2,269,154
20–84	PJI (hip)	novel species **	PNS	*appendicis* (45)	sp017942225	-	62.7	2,408,199
17–2097	PSII	*suicordis*	*suicordis*	*suicordis* (74)	*suicordis*	96.73	60.1	2,282,974
21–2754	PJI (hip)	*marquesiae*	PNS	*marquesiae* (76)	*marquesiae*	96.20	59.3	2,533,370
17–3561-b	PJI (hip)	*marquesiae*	PNS	*marquesiae* (76)	*marquesiae*	96.15	59.2	2,591,729
21–1018	PSII	*marquesiae*	*marquesiae*	*marquesiae* (75)	*marquesiae*	96.45	59.4	2,464,461
21–725	Same patient as above	*marquesiae*	*marquesiae*	*marquesiae* (75)	*marquesiae*	96.45	59.4	2,464,381
18–6638	Osteitis (after amputation)	*tuberculostearicum*	PNS	*tuberculostearicum* (100)	*tuberculostearicum*_F	95.91	59.9	2,450,981
20–1674	Osteitis (hand)	*tuberculostearicum*	PNS	*tuberculostearicum* (95)	no hit	95.80	59.4	2,643,437
23–591	PSII	marquesiae *	PNS	*marquesiae* (76)	*marquesiae*	94.98	59.6	2,458,070
23–592	Same patient as above	marquesiae *	PNS	*marquesiae* (76)	*marquesiae*	94.99	59.6	2,460,178
18–74	PSII	*marquesiae*	PNS	*marquesiae* (76)	*marquesiae*	96.16	59.3	2,533,069
23–383	Osteitis (humerus, fracture)	*marquesiae*	*marquesiae*	*marquesiae* (89)	*marquesiae*	96.50	59.5	2,481,110
19–1446	Keratitis	*uberis*	PNS	*uberis* (100)	*uberis*	99.95	65.6	2,503,313
19–1445	Same patient as above	*uberis*	PNS	*uberis* (100)	*uberis*	99.94	65.6	2,503,940
17–3561-a	PJI (hip)	*mucifaciens*	*mucifaciens*	*mucifaciens* (49)	*mucifaciens*	97.49	65.8	2,086,028
16–7024	PSII	*mucifaciens*	*mucifaciens*	*mucifaciens* (53)	*mucifaciens*	97.57	65.7	2,135,243

*Abbreviations*: *CSF* cerebrospinal fluid, *PJI* prosthetic joint infection, *PSII* postoperative spinal implant infection, *SSI* surgical site infection, *PNS* potential novel species

*isolates with an ANI of 92% - 95% to the reference genome of the nearest known relative (Table S2)

**isolates with an ANI of <90% to the reference genome of the nearest known relative (Table S2)

### Genomic diversity of clinical corynebacterial isolates

All 56 isolates were genome sequenced. Genome statistics are listed in Table 1 and Table S1. The GC content of the genomes varied substantially: 56.1% (23–3061) to 72.8% (23–137 and 23–416), with a mean GC content of 61.8%. The genome size varied drastically, from 2,086 kb (17-3561_a) to 2,912 kb (23–692), with a mean of 2,486 kb. Species identification based on genome sequences was done with multiple tools, including TYGS [37], rMLST [38] and GTDB-tk [39], resulting in 28 identified species. Many isolates could not unambiguously be assigned to a known species (Table 1). Additionally, the 56 genomes were compared by genome-wide average nucleotide identity (ANI) calculations to reference genomes of their closest relatives (Table S2). Taken together, 43 of 56 isolates could be assigned to a known species, with an ANI of > 95% to the respective reference genome, which is often used as threshold for species assignment [46]. Another eleven isolates could be closely linked to a species, having an ANI of 92–95% to the closest known relative. Two isolates likely represent novel species: isolates 20–84 and 22–2729 had no close relative; highest ANIs were detected with the reference genome of *C. appendicis* (86.59%) and *C. jeikeium* (83.08%), respectively (Table S2).

**Fig. 1:**
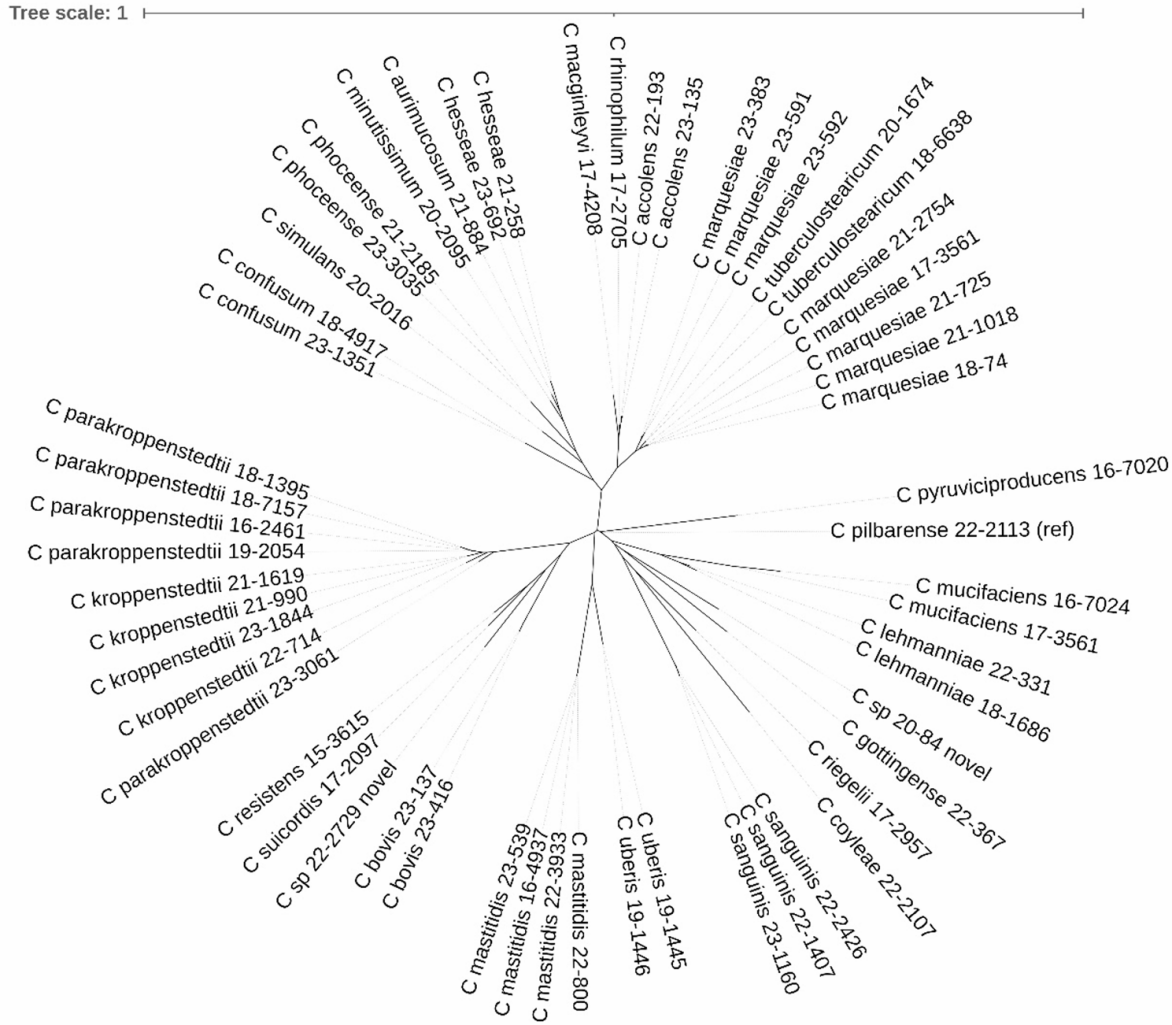
Core genome-based phylogeny of the sequenced corynebacterial strains. In this study, 56 strains isolated from diverse clinical specimens were compared. Core genome alignment was done with CSI phylogeny. A total of 5542 reliable single nucleotide variants (SNVs) were identified in the core genome and used for phylogenetic reconstruction. Strain *C. pilbarense* 22–2113 was used as reference

A phylogenetic tree based on core genome comparison was built, showing the diversity of the 56 isolates (Fig. 1). Several species were closely related and form clades/complexes, including *C. tuberculostearcium* - *C. marquesiae; C. kroppenstedtii - C. parakroppenstedtii*; *C. aurimucosum* - *C. hesseae* - *C. minutissimum*, and *C. accolens* - *C. macginleyi* - *C. rhinophilum*. Their kinship was also reflected in ANIs of 87–95% (Table S2). A further phylogenetic comparison with *Corynebacterium* spp. reference genomes underlined the high diversification (Fig. S1). Although most strains were unique and were individual strains, a few strain pairs were identified as highly similar based on ANI and phylogenetic analyses. We hypothesized that these strain pairs belonged to the four patient samples, for which two strains each were sequenced. To clarify this, a single nucleotide variant (SNV) analysis was performed and clonality was defined as the presence of no or very few SNVs (≤ 20) in the core genomes (Fig. S2). It could be confirmed that all four strain pairs derived from the same patient materials were clonal: the *C. marquesiae* strain pairs 23–591 and 23–592 (PSII) and 21–725 and 21–1018 (PSII); the *C. kroppenstedtii* strain pair 21–990 and 21–1619 (shunt infection) and the *C. uberis* strain pair 19–1445 and 19–1446 (keratitis). Interestingly, in addition to these four pairs a few other strains were found to be clonal or highly similar: *C. marquesiae* strains 18–74 and 17-3561_b were clonal, but originated from two distinct patients with a spinal infection and PJI, respectively. Regarding this pair, another *C. marquesiae* strain, 21–2754 (PJI patient), was highly similar (43 SNVs). The two *C. parakroppenstedtii* strains 18–7157 and 18–1395 were highly similar (24 SNVs); these strains originated from two distinct patients with mastitis.

### Morphological and metabolic differences among clinical corynebacteria

**Fig. 2:**
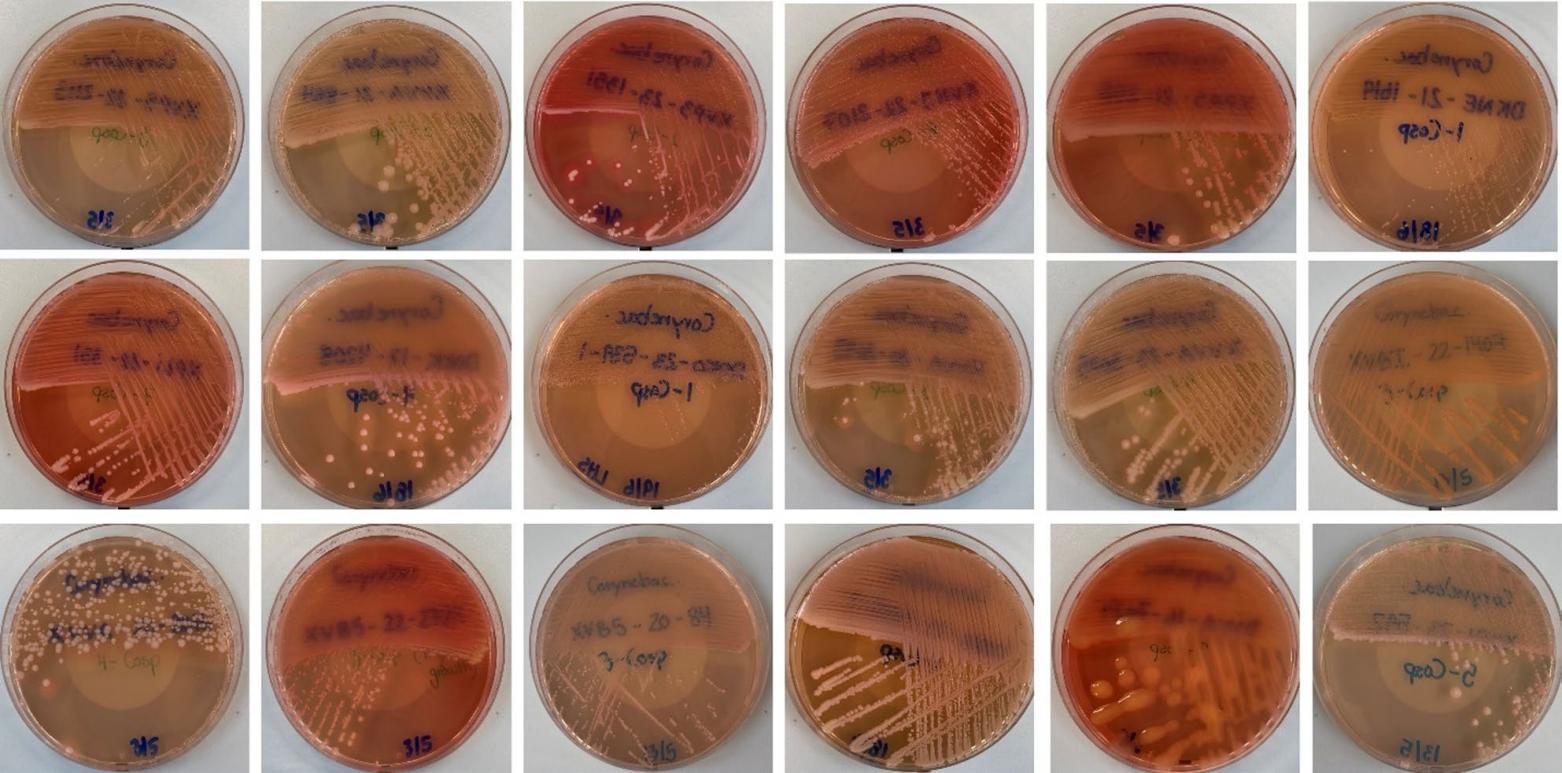
Colony morphology of corynebacterial strains on FTO agar medium. Plate pictures of colonies of 18 strains belonging to 18 different species are depicted. Row 1 (left to right): *C. pilbarense* 22–2113; *C. aurimucosum* 21–884; *C. confusum* 23–1351; *C. coyleae* 22–2107; *C. hesseae* 21–258; *C. kroppenstedtii* 21–1619. Row 2: *C. lehmanniae* 22–331; *C. macginleyi* 17–4208; *C. mastitidis* 23–539; *C. minutissimum* 20–2095; “*C. phoceense”* 23–3035; *C. sanguinis* 22–1407. Row 3: *C. simulans* 20–2016; *C*. sp. 22–2729; *C*. sp. 20–84; *C. uberis* 19–1445; *C. mucifaciens* 16–7024; *C. marquesiae* 23–592

It was noticed that colony morphology of the 56 corynebacterial strains on FTO agar was very variable, ranging from tiny colonies to large mucoid viscous colonies (Fig. 2). In addition, colony opacity and color varied, from translucent to opaque and from white/beige to yellow and orange-red, respectively.

Next, we determined and compared biochemical properties of the corynebacterial isolates. 34 representative strains belonging to 22 species were selected, covering the genomic diversity reflected in the phylogenetic tree (Fig. 1). Biochemical tests (API™ Coryne) were performed to test enzymatic activities and sugar-degrading capacities (Table 2). All 34 strains were negative for the following reactions: enzymatic activity of β-galactosidase, N-acetyl-β-glucosaminidase, and gelatinase, and fermentation of the sugars xylose, lactose, and glycogen. Most strains were negative regarding the presence of α-glucosidase (2/34), β-glucuronidase (3/34), urease (5/34) and β–glucosidase (10/34) activity, fermentation of the sugar mannitol (3/34), and reduction of nitrate (7/34). Most strains were positive for pyrazinamindase (31/34) and alkaline phosphatase (28/34) activity and the fermentation of glucose (27/34). A large variation existed between different species, e.g. the two *C. uberis* strains were positive in 10/19 tests, whereas the *C. confusum* strains were only positive in 3/19 tests. To investigate the genomic basis of their enzymatic activities, genomes were searched for nitrate reductases and ureases (Fig. S3): 10/34 strains, including the species *C. confusum*,* C. accolens*,* C. rhinophilum*,* “C. phoceense”*,* C. uberis* and *C. pyruviciproducens* contained the gene cluster for a respiratory nitrate reductase (Fig. S3A); 7/34 strains harbored the genes for urease, including *C. mastiditis*,* C. uberis* and *C. parakroppenstedtii* (Fig. S3B).

**Table 2:**
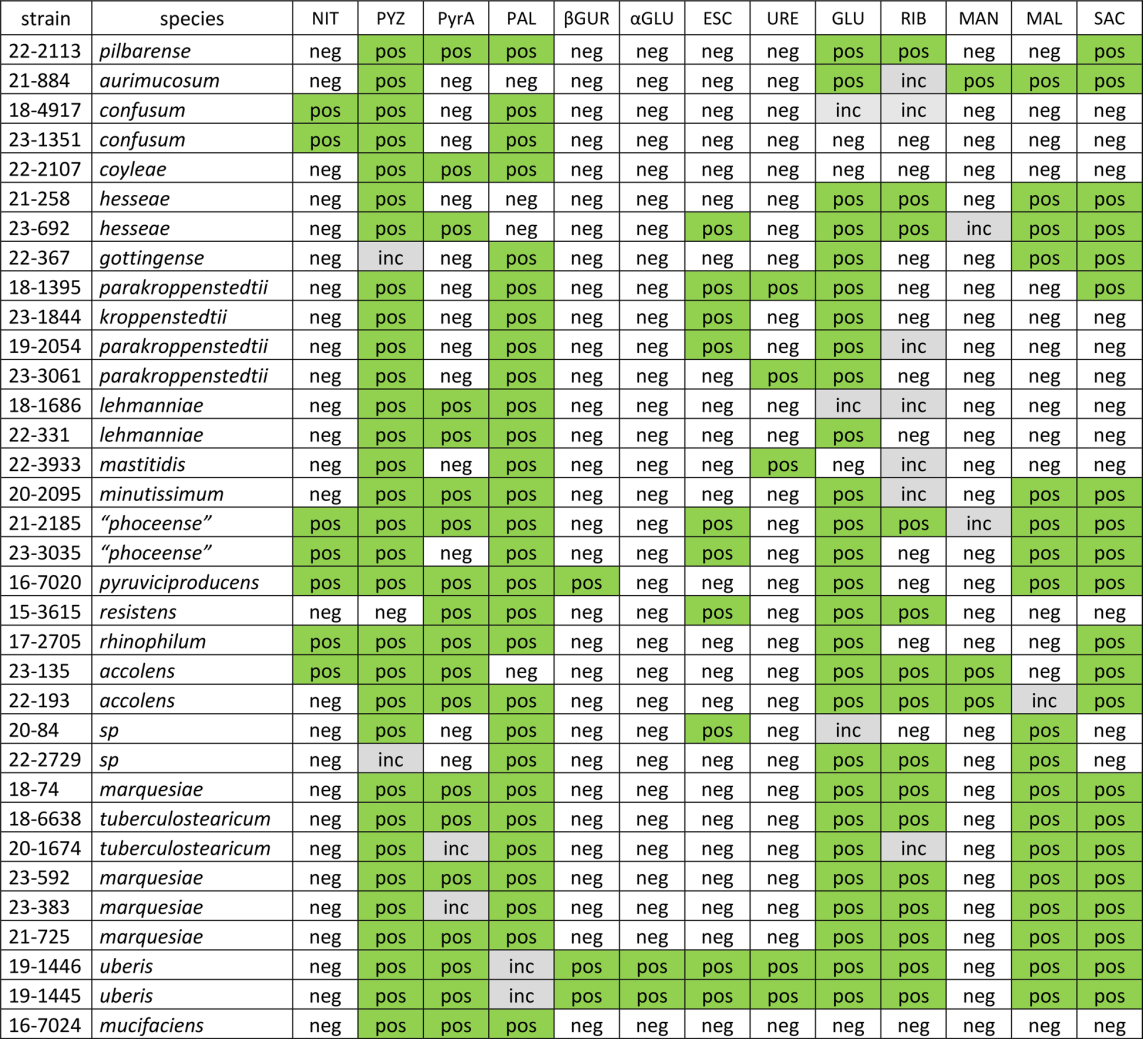
Biochemical properties of the 34 corynebacterial isolates

*Abbreviations*: *NIT* (nitrate reduction), *PYZ* (pyrazinamindase), *PyrA* (pyrrolidonyl acrylamidase), *PAL* (alkaline phosphatase), *βGUR* (β-glucuronidase), *αGLU* (α-glucosidase), *ESC* (β–glucosidase, esculin), *URE* (urease); fermentation of glucose (GLU), ribose (RIB), mannitol (MAN), maltose (MAL), and saccharose (SAC), *inc* inconclusive

### Extended resistance to clindamycin, and multiple resistances in some clinical corynebacteria

Antimicrobial susceptibility testing (AST) using the disc diffusion assay (DDA) was performed for the same 34 isolates that have been selected also for biochemical testing. The following antibiotics were tested: penicillin, clindamycin, ciprofloxacin, vancomycin and rifampicin (Table 3). According to the EUCAST guidelines, MH-F plates (Mueller-Hinton agar + 5% defibrinated horse blood and 20 mg/L β-NAD) should be used for *Corynebacterium* spp. In contrast to FTO agar, we noticed very poor growth on MH-F agar of several species/isolates, including *C. marquesiae* 23–592, 23–383 and 21–725, *C. parakroppenstedtii* 19–2054, *C. accolens* 22–193 and *C.* sp. 22–2729, which made zone diameter determination difficult (Fig. S4). To test if DDA could be performed on FTO instead of MH-F agar, we performed DDA for eight strains simultaneously on MH-F and FTO agar. Similar results regarding zone diameters were obtained (Table S3).

**Table 3:** Antimicrobial susceptibility testing of 34 corynebacterial isolates against five antibiotics

strain	species	Ciprofloxacin	Clindamycin	Penicillin	Rifampicin	Vancomycin
22-2113	*pilbarense*	-	+ (NZ)	-	-	-
21-884	*aurimucosum*	IM	+ (NZ)	IM	-	-
18-4917	*confusum*	IM	-	IM	-	-
23-1351	*confusum*	IM	-	IM	-	-
22-2107	*coyleae*	IM	+	IM	-	-
21-258	*hesseae*	+ (NZ)	+	IM	-	-
23-692	*hesseae*	+ (NZ)	+ (NZ)	+	-	-
22-367	*gottingense*	-	-	IM	-	-
18-1395	*parakroppenstedtii*	-	+ (NZ)	IM	-	-
23-1844	*kroppenstedtii*	-	-	IM	-	-
19-2054	*parakroppenstedtii*	+ (NZ)	+ (NZ)	IM	-	-
23-3061	*parakroppenstedtii*	-	-	IM	-	-
18-1686	*lehmanniae*	+	+ (NZ)	IM	-	-
22-331	*lehmanniae*	+ (NZ)	+ (NZ)	+ (NZ)	-	-
22-3933	*mastitidis*	-	-	-	-	-
20-2095	*minutissimum*	IM	+	IM	-	-
21-2185	*“phoceense”*	IM	+ (NZ)	IM	-	-
23-3035	*“phoceense”*	IM	+ (NZ)	IM	-	-
16-7020	*pyruviciproducens*	IM	-	-	-	-
15-3615	*resistens*	IM	+ (NZ)	+	-	-
17-2705	*rhinophilum*	-	-	IM	-	-
23-135	*accolens*	-	-	IM	-	-
22-193	*accolens*	IM	+	IM	-	-
20-84	*sp*	IM	+	IM	-	-
22-2729	*sp*	IM	+	+	-	-
18-74	*marquesiae*	+ (NZ)	+ (NZ)	+ (NZ)	+	-
18-6638	*tuberculostearicum*	IM	+	IM	-	-
20-1674	*tuberculostearicum*	+ (NZ)	+ (NZ)	+ (NZ)	-	-
23-592	*marquesiae*	+ (NZ)	+ (NZ)	+ (NZ)	+	-
23-383	*marquesiae*	+ (NZ)	+	IM	-	-
21-725	*marquesiae*	+ (NZ)	+ (NZ)	+ (NZ)	+	-
19-1446	*uberis*	-	-	-	-	-
19-1445	*uberis*	-	-	-	-	-
16-7024	*mucifaciens*	-	+ (NZ)	IM	-	-

*Abbreviations*: + resistant, - susceptible, *IM* intermediate, *NZ* no inhibition zone

AST results showed that all strains were susceptible to vancomycin and most were susceptible to rifampicin (31/34). Resistance to clindamycin was most prevalent with 23/34 resistant strains. Penicillin and ciprofloxacin resistance was also extensive with 29/34 and 23/34 fully or intermediate resistant strains, respectively. Extended multi-resistance was detected in strains of *C. marquesiae*,* C. tuberculostearicum*,* C. lehmanniae*,* C. hesseae* and *C. resistens.*

Looking at the genomic basis of the identified resistances, *erm(X)*, encoding a 23S rRNA methyltransferase was found in 15/34 strains, including the strains of the species *C. pilbarense*,* C. aurimucosum*,* C. hesseae*,* C. gottingense*,* C. parakroppenstedtii*,* C. lehmanniae*,* “C. phoceense”*,* C. tuberculostearicum*,* C. marquesiae* and *C. mucifaciens* (Table S4). This gene is associated with macrolide-lincosamide resistance, including resistance to clindamycin [47–49]. The *C. marquesiae* strain 21–725 which was found to be resistant to all tested antibiotics except vancomycin harbors seven AMR genes, including *erm(X)*, four genes for aminoglycoside inactivation by either phosphorylation (*aph*(3’)-Ia, *aph*(3’’)-Ib, *aph*(6)-Id) or acetylation (*aac(3)-XI*), *cmx* (chloramphenicol efflux pump) and *tet(W)* (ribosomal protection protein).

## Discussion

This study investigated *Corynebacterium* isolates from clinical specimens that could not unambiguously be assigned to a specific species due to a low MALDI TOF MS identification score. Genome sequencing and comparison to genome sequence databases showed that most of these isolates were relatives to known corynebacterial species, except for two isolates that likely represent undescribed species. These findings reflect two issues with MALDI TOF MS-based species identification:

(I) The MALDI TOF MS database can lack rare corynebacterial species and/or those that are so far undescribed or have only recently been identified. In the last years several novel human-associated corynebacterial species have been described, including *C. senegalense* [50], *C. hindlerae* [51], *C. guaraldiae* [52], *C. lehmanniae*, *C. meitnerae*, *C. evansiae*, *C. curieae*, *C. macclintockiae*, *C. hesseae*, *C. marquesiae*, *C. yonathiae* [53], *C. parakroppenstedtii*, *C. pseudokroppenstedtii* [54], “*C. hallux”*,* “C. nasorum”* [55], *“C. vikingii”*, “*C. borealis”* [6], “*C. axilliensis”* and “*C. jamesii”* [56]. Results from metagenomic sequencing of human skin suggests the existence of other rare corynebacterial species [4, 5, 57]. As MALDI TOF MS database updates require some time, it is likely that some corynebacterial species will not be (correctly) identified, as observed in this study. The two undescribed species found here had some relatedness to *C. appendicis* and *C. jeikeium*, respectively.
(II) The MALDI TOF MS database covers a species often only with one (i.e., the type strain of a given species) or few isolates. Most sequenced strains here exhibited substantial differences from the genomes of reference strains, as reflected by low ANI values. Thirteen out of 56 strains showed ANI values below the 95% threshold, and another 21 strains had an ANI between 95–97% to the genome of the closed reference strain. Low MALDI TOF MS identification scores thus mirror these low ANI values. To take extensive strain variation within a given species into account in MS-based species identification, a more diverse representation of strains per species should ideally be included in the MS database.

Besides these MALDI TOF MS issues, our findings raised also another challenge, i.e. the concept of species boundary definition. Current DNA homology-based taxonomic definitions suggest an ANI cutoff of 95% to define species boundaries [46]. However, here ANI values close to and below 95% were often identified. We argue here that it does not make much sense to assign new species names to strains that have ANI values in the close vicinity of 95%. For example, it is questionable to distinguish *C*. *marquesiae* from *C. tuberculostearicum* as the ANI between some *C*. *marquesiae* strains are close to 95% (e.g., ANI of 95.41% for *C*. *marquesiae* strains 23–592 and 23–383), and similar ANIs exist between some strains of *C. tuberculostearicum* and *C*. *marquesiae* (e.g., ANI of 94.55% for strains *C. tuberculostearicum* 18–6638 and *C*. *marquesiae* 23–592), which is also reflected in the phylogenomic tree (Fig. S1). This was also described in other recent studies [5, 58]. We suggest assigning the *C*. *marquesiae/C. tuberculostearicum* strains to the *C. tuberculostearicum* complex. This also applies for *C. kroppenstedtii-C. parakroppenstedtii*, as recently discussed [59]. Some *C. kroppenstedtii* strains had an ANI below 95% between each other (e.g., ANI of 91.74% for strains 21–990 and 22–714); likewise, some *C. parakroppenstedtii* had a low ANI between each other (e.g. ANI of 91.78% for strains 16–2461 and 23–3061). There was a recent discussion on the limited usefulness of a strict ANI threshold for species boundary definition [60]. The authors argued regarding commensal streptococci (*Streptococcus mitis* and *Streptococcus oralis*) that classifying most isolates as separate species has little biological or practical value as *S. mitis* and *S. oralis* form a continuous range of genetically distinct clones. They suggested adjusting the current similarity thresholds to better reflect the true biological relationships. This could also be applied to commensal corynebacteria to limit the appearance of many new species names. However, new species names should be reserved for strains with significant functional differences or specific clinical associations with disease.

What is the clinical significance of these *Corynebacterium* strains identified in human specimens? Many strains originated from specimens obtained from medical device-related infections, such as PJI and shunt implant infections, e.g. seven of the ten *C. tuberculostearicum* complex strains were isolated from PSII or PJI. It could not be verified here if these isolates were the source of infection or were passive bystanders or contaminants during the sample taking process, but the isolated corynebacteria were the predominant organisms upon cultivation of the clinical specimens included in this study. It is likely that at least some of the here identified and sequenced corynebacteria were causative agents of infection. This adds to current assumptions that *Corynebacterium* sp. could be an underestimated pathogen [61]. It is furthermore noteworthy that some strains exhibited an extensive antibiotic resistance profile, particularly within the *C. tuberculostearicum* complex: four out of the six tested strains were resistant to at least four antibiotics. Due to the lack of a comparison group (e.g., *Corynebacterium* sp. from healthy skin), it remains unclear whether these resistances were endogenous or acquired during treatment.

This study has several limitations. First, a non-standard AST/DDA approach was used because some *Corynebacterium* strains did not grow sufficiently on the EUCAST-recommended MH-F agar. In addition, only a limited range of antibiotics was tested; notably, no aminoglycosides were included, which should be incorporated in future work. We also did not perform species-specific core-genome analyses, as the number of isolates per species was small. Future studies with larger sample sizes could explore within-species genomic diversity and link it to strain-specific phenotypic traits. For ANI calculations, we used reference genomes that were not always identical to the type strain genomes. Finally, comprehensive clinical data for the included infection cases were unavailable, making any assessment speculative of how likely the isolated strains were to be the causative agents.

## Conclusions

In conclusion, this study enhances our understanding of *Corynebacterium* strains isolated from clinical samples, especially those that deviate from established type/reference strains. It highlights the significant genetic and phenotypic diversity present within many *Corynebacterium* species and questions the adequacy of strict ANI thresholds for defining species boundaries. Additionally, at least two previously undescribed *Corynebacterium* species were identified that warrant further characterization. The genome data generated in this work provide a valuable foundation for future research into the pathogenic properties and clinical significance of these isolates.

## Supplementary Information

Supplementary Material 1.

Supplementary Material 2.

Supplementary Material 3.

Supplementary Material 4.

Supplementary Material 5.

## Data Availability

The genome sequence data are available in the GenBank repository, with the following accession number: PRJNA1327770. The data can be accessed here: https://www.ncbi.nlm.nih.gov/bioproject/PRJNA1327770/.

## Abbreviations

AMR: antimicrobial resistance
ANI: average nucleotide identity
AST: antimicrobial susceptibility testing
DDA: disc diffusion assay
FTO: Furazolidone, Tween-80, Oil red O
MH-F: Mueller-Hinton agar + 5% defibrinated horse blood and 20 mg/L β-NAD
PJI: prosthetic joint infection
PSII: postoperative spinal implant infection
SNV: single nucleotide variant
SSI: surgical site infection
UTI: urinary tract infection
