# Supplementary material for: Genomic and phenotypic diversity among taxonomically ambiguous clinical Corynebacterium isolates
Source: BMC Microbiol. 2025 Dec 22;26:60. doi: 10.1186/s12866-025-04619-8 (PMC12849385; doi:10.1186/s12866-025-04619-8)
Supplement: Supplementary file 1 — Supplementary Material 1. [file 12866_2025_4619_MOESM1_ESM.pdf]

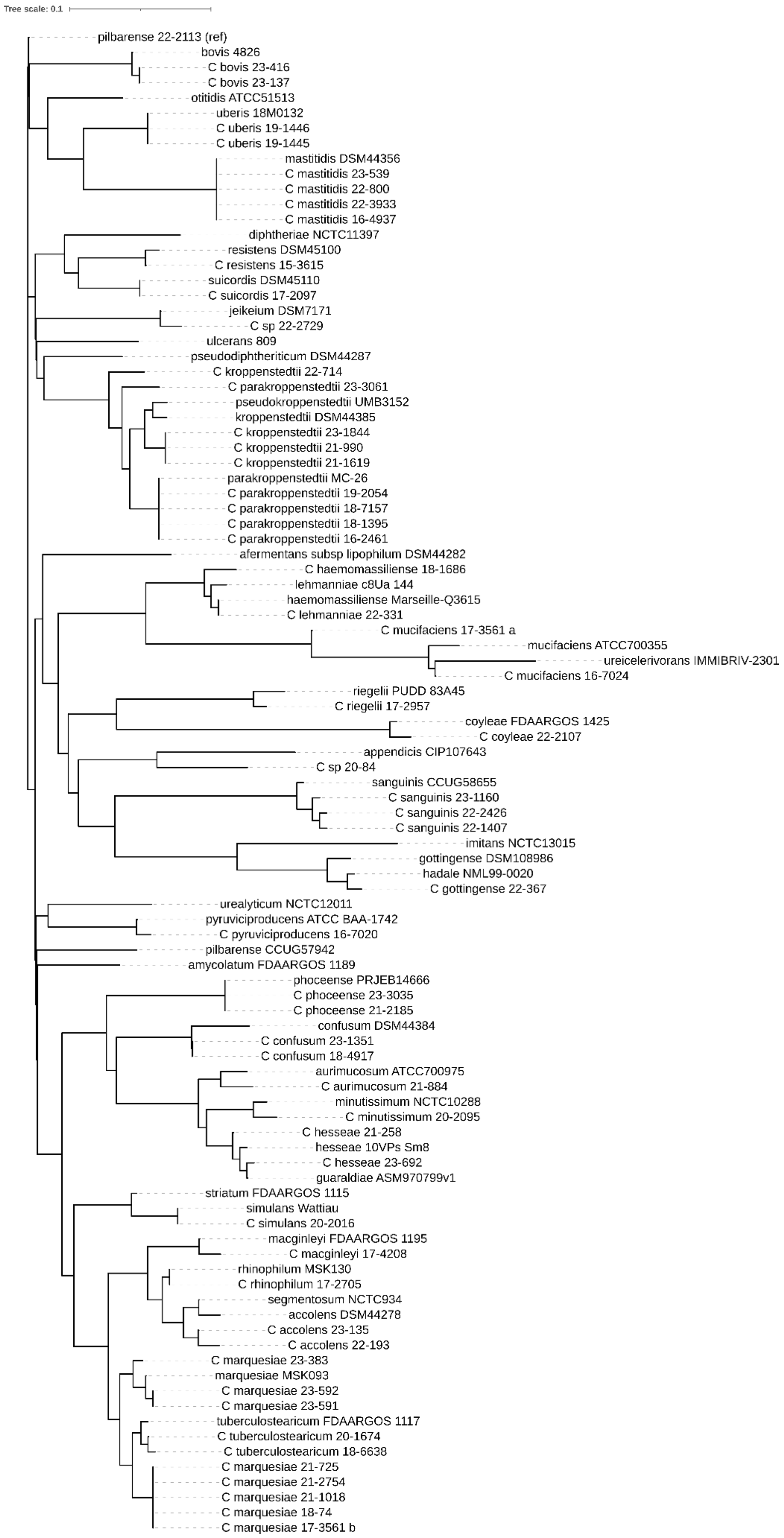
Tree scale: 0.1
pilbarense 22-2113 (ref)
bovis 4826
C bovis 23-416
C bovis 23-137
otitidis ATCC51513
uberis 18M0132
C uberis 19-1446
C uberis 19-1445
mastitidis DSM44356
C mastitidis 23-539
C mastitidis 22-800
C mastitidis 22-3933
C mastitidis 16-4937
diphtheriae NCTC11397
resistens DSM45100
C resistens 15-3615
suicordis DSM45110
C suicordis 17-2097
jeikeium DSM7171
C sp 22-2729
ulcerans 809
pseudodiphtheriticum DSM44287
C kroppenstedtii 22-714
C parakroppenstedtii 23-3061
pseudokroppenstedtii UMB3152
kroppenstedtii DSM44385
C kroppenstedtii 23-1844
C kroppenstedtii 21-990
C kroppenstedtii 21-1619
parakroppenstedtii MC-26
C parakroppenstedtii 19-2054
C parakroppenstedtii 18-7157
C parakroppenstedtii 18-1395
C parakroppenstedtii 16-2461
afermentans subsp lipophilum DSM44282
C haemomassiliense 18-1686
lehmanniae c8Ua 144
haemomassiliense Marseille-Q3615
C lehmanniae 22-331
C mucifaciens 17-3561 a
mucifaciens ATCC700355
ureicelerivorans IMMIBRIV-2301
C mucifaciens 16-7024
riegelii PUDD 83A45
C riegelii 17-2957
coyleae FDAARGOS 1425
C coyleae 22-2107
appendicis CIP107643
C sp 20-84
sanguinis CCUG58655
C sanguinis 23-1160
C sanguinis 22-2426
C sanguinis 22-1407
imitans NCTC13015
gottingense DSM108986
hadale NML99-0020
C gottingense 22-367
urealyticum NCTC12011
pyruviciproducens ATCC BAA-1742
C pyruviciproducens 16-7020
pilbarense CCUG57942
amycolatum FDAARGOS 1189
phoceense PRJEB14666
C phoceense 23-3035
C phoceense 21-2185
confusum DSM44384
C confusum 23-1351
C confusum 18-4917
aurimucosum ATCC700975
C aurimucosum 21-884
minutissimum NCTC10288
C minutissimum 20-2095
C hesseae 21-258
hesseae 10VPs Sm8
C hesseae 23-692
guaraldiae ASM970799v1
striatum FDAARGOS 1115
simulans Wattiau
C simulans 20-2016
macginleyi FDAARGOS 1195
C macginleyi 17-4208
rhinophilum MSK130
C rhinophilum 17-2705
segmentosum NCTC934
accolens DSM44278
C accolens 23-135
C accolens 22-193
C marquesiae 23-383
marquesiae MSK093
C marquesiae 23-592
C marquesiae 23-591
tuberculostearicum FDAARGOS 1117
C tuberculostearicum 20-1674
C tuberculostearicum 18-6638
C marquesiae 21-725
C marquesiae 21-2754
C marquesiae 21-1018
C marquesiae 18-74
C marquesiae 17-3561 b

Supplementary Figure S1: Core genome-based phylogeny of corynebacteria sequenced in this study together with reference genomes of corynebacterial strains. 56 strains isolated from clinical specimens (Table 1), and 43 reference genomes of closely related species were compared. Core genome alignment and SNV identification was done with CSI phylogeny. 2299 reliable SNVs were identified in the core genome and used for phylogenetic reconstruction. Strain *C. pilbarense* 22-2113 was used as reference.

A

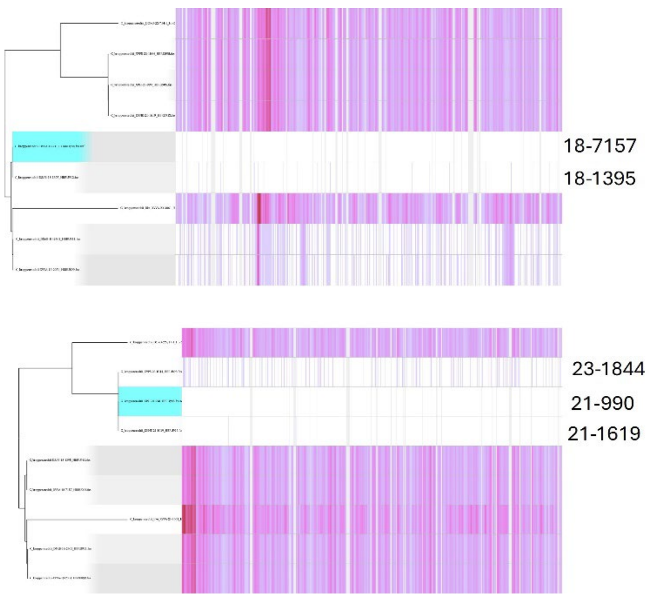

B

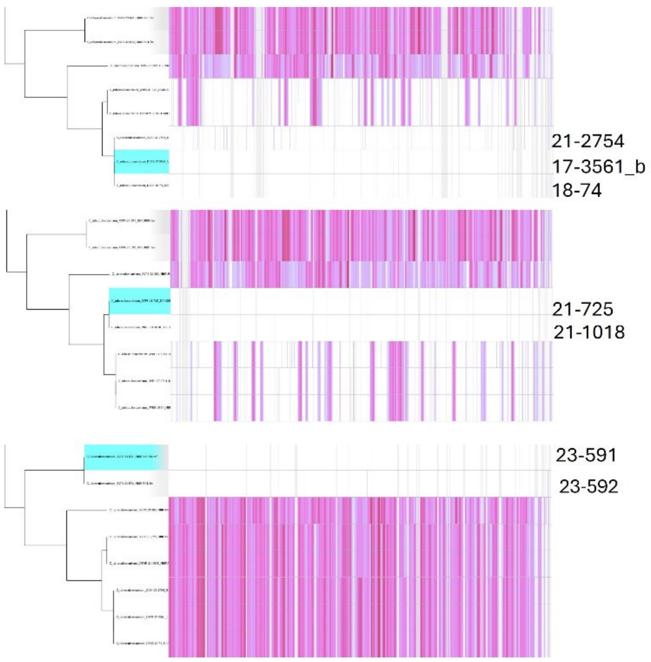

Supplementary Figure S2: Single nucleotide variant analysis of closely related corynebacterial genomes. Core genome alignment and single nucleotide variant (SNV) analysis were done with ParSNP. Clonality was defined as <= 5 SNVs between the core genomes; high relation was defined as 5 < SNVs < 50. A) Strains of *C. kroppenstedtii/C. parakroppenstedtii* were compared. One clonal pair (*C. kroppenstedtii* 21-990 and 21-1619; 2 SNVs) and one highly related pair (*C. parakroppen-stedtii* 18-7157 and 18-1395; 24 SNVs) were identified. B) Strains of *C. marquesiae* were com-pared; three clonal pairs (*C. marquesiae* 23-591 and 23-592 (5 SNVs); 21-725 and 21-1018 (2 SNVs); 18-74 and 17-3561_b (3 SNVs)) were found. One additional strain (*C. marquesiae* 21-2754) is highly related (43 SNVs) to one clonal pair (18-74 and 17-3561_b).

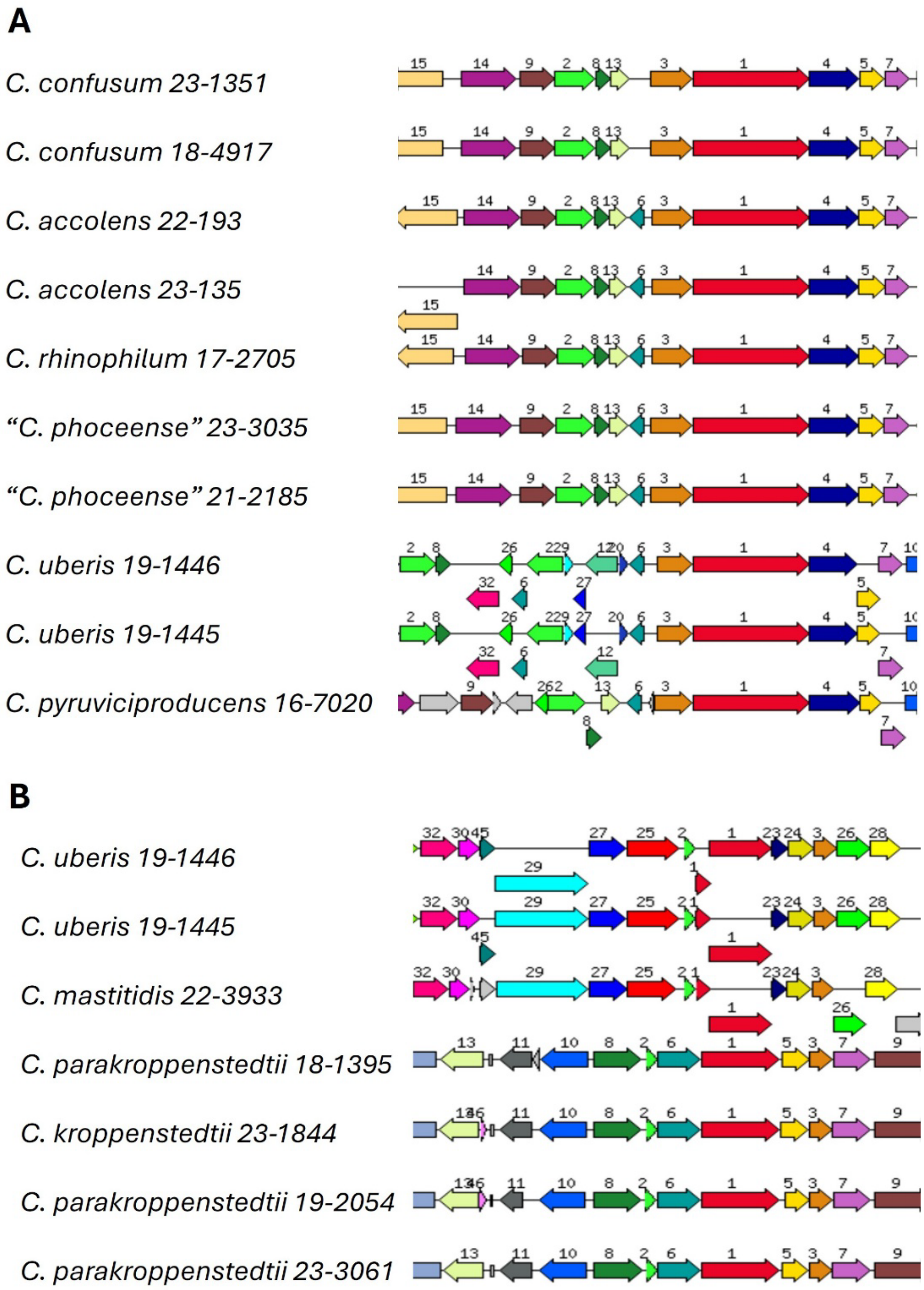

Supplementary Figure S3: Gene clusters in corynebacteria encoding respiratory nitrate reductase and urease. A. Respiratory nitrate reductase. Numbers show specific subunits and related genes: 1, nitrate reductase alpha chain; 3, nitrate/nitrite transporter NarK/U; 4, nitrate reductase beta chain; 5, nitrate reductase delta chain; 7, nitrate reductase gamma chain. B. Urease. 1, Urease alpha subunit; 2, Urease gamma subunit; 3, Urease accessory protein UreG; 7 and 26, Urease accessory protein UreD; 5 and 24, Urease accessory protein UreF; 6 and 23, Urease accessory protein UreE.

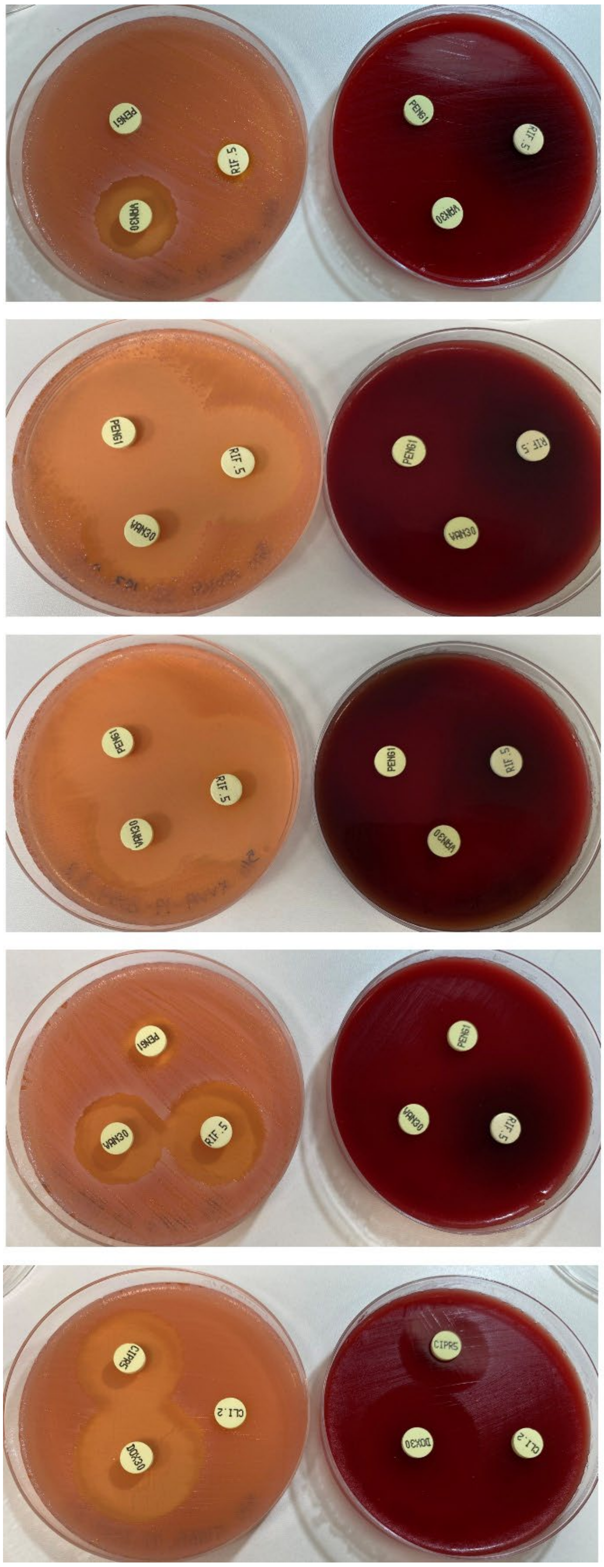

21-725

22-193

19-2054

23-383

22-2729

Supplementary Figure S4: Disc diffusion assay of *Corynebacterium* strains grown on FTO agar and MH-F agar. Growth on FTO (left) was better than growth on MH-F (right). Zone diameters on MH-F agar were difficult to measure for some *Corynebacterium* strains.
